# Treatment of Malignant Liver Tumors by Radiofrequency Ablation Combined with Low-Frequency Ultrasound Radiation with Microbubbles

**DOI:** 10.1371/journal.pone.0053351

**Published:** 2013-01-10

**Authors:** Ai Junhua, Jin Yun, Wang Zhenzhou, Yang Ling, Luo Ding

**Affiliations:** 1 Department of General Surgery, Chinese People's Armed Police Force 8710 Hospital, Putian, Fujian Province, People's Republic of China; 2 Department of Hepatobiliary Surgery, Kunming General Hospital Affiliated Chengdu Military District of Chinese People's Liberation Army, Kunming, Yunnan Province, People's Republic of China; 3 Deparment of Medical Engineering, Kunming General Hospital Affiliated Chengdu Military District of Chinese People's Liberation Army, Kunming, Yunnan, Province, People's Republic of China; 4 Department of Special Diagnosis, Kunming General Hospital Affiliated Chengdu Military District of Chinese People's Liberation Army, Kunming, Yunnan Province, People's Republic of China; University of Navarra School of Medicine and Center for Applied Medical Research (CIMA), Spain

## Abstract

**Objective:**

To explore the therapeutic efficacy and safety of malignant liver tumor treatment by radiofrequency ablation (RA) combined with low frequency ultrasound radiation with microbubbles (LFURM).

**Methods:**

Retrospective analysis of 25 patients with malignant hepatic tumors treated by RA/LFURM in the Department of Hepatobiliary Surgery of Kunming General Hospital affiliated to Chengdu Military District, PLA from January 2010 to June 2011. Ultrasound guided RA was performed, which was followed one week later by LFURM. Basal contrast ultrasound, liver function tests, and serum alpha fetoprotein (AFP) were obtained, and repeated 3 and 6 months after treatment. T-test and chi-square were used to compare parametric and non-parametric variables respectively.

**Results:**

In 17 cases, gross tumor volume was significantly reduced 6 months after treatment while mean tumor showed a reduction of 50% compared to pre-treatment values. In 7 cases gross tumor volumes reduction was partial, but surrounding tumor tissue showed blood flow signals. One patient had no reduction in gross tumor volume. Levels of serum alanine aminotransferase (ALT), aspartate aminotransferase (AST), total bilirubin (TBIL), alpha fetoprotein (AFP) decreased significantly 6 months after treatment (all *p*<0.05). No tumor recurrence was seen during the 6 month follow-up. Quality of life scores (QOL) were good in 21 patients (84%), improved in 2 patients (8%), unchanged in 1 patient (4%) and got worst in 1 patient (4%). Karnofsky scores (KPS) improved in 19 patients (76%), remained unchanged in 5 patients (2%) and got worst in 1 patient (4%). Both QOL and KPS changes were statistically significant (*P*<0.05).

**Conclusion:**

RA/LFURM treatment of liver tumors is efficient and safe, and can reduce the gross tumor volumes and protect liver function.

## Introduction

Over fifty percent of liver cancer victims live in China. Surgical resection is the most frequent form of treatment for malignant liver tumors. However, in most cases, tumors are detected in late stages when liver function is already very poor. Radical resection can be performed in only 30% of cases which makes recurrence rates very high [1], [2]. Although a second resection can be made for some recurrent tumors, in most cases only one resection can be performed. If possible, hepatic surgeons should select minimally invasive procedures with good therapeutic results in order to preserve the best possible liver function. Radiofrequency ablation (RA) is a minimally invasive technique done through a small wound, and can efficiently destroy malignant tissue and transfer heat through surrounding large vessels, but tumor cells can remain and can induce recurrence. For that reason RA has limited use [3], [4]. Low frequency ultrasound radiation with microbubbles (LFURM) is a new technique used in the treatment of malignant tumors, which erodes the tissue by cavitation, affecting micro-vessels producing ischemia and necrosis of tumor tissue. However, the process is slow and does not affect large vessels [5], [6]. To combine the benefits and overcome the deficiencies of those techniques, they were used in close succession in 25 patients with malignant liver tumors between January, 2010 and June, 2011.

## Patients and Methods

### Patients

Of the 25 patients with malignant liver tumors 9 were females and 16 were males. Ages ranged from 23 to 78 years (mean age, 53.3 years). Liver function (Child-Pugh classification) was class A in 15 patients, class B in 7 patients and class C in 3 patients (which improved after medical treatment). Tumor stage was I in 9 patients and II–IV in 16 patients (American Cancer Society). Primary hepatic cancer (PHC) was present in 15 cases and recurrent liver tumor in 10 cases. Hepatocellular carcinoma (HCC) was found in 22 cases and in 3 cases the type was cholangiocellular carcinoma (CC). AFP was positive in 21 cases. Percutaneous contrast ultrasound was performed in all patients before surgery. All tumors were more than 3.0 cm in diameter and in 3 cases the tumors were larger than 5 cm in diameter. More than one tumor were present in 23 cases. There was no extrahepatic spread of the tumor in any of the patients. Data were gathered from a retrospective review of medical charts with approval from the Institutional Review Board.

#### Inclusion and exclusion criteria

Inclusion criteria: (1)Age between 18–80 years old; (2)Tumor lesion could be clearly determined by percutaneous contrast ultrasound; (3)Tumor size between 3–8 cm in diameter; (4)The maximum number of tumors was 3; (5)Histopathological diagnosis (HCC or CC) was established by ultrasound guided liver biopsy: (6)No history of previous malignant disease;(7)Patients did not receive any other treatment in the last 3 months.

Exclusion criteria: (1)Patients with liver malignant tumor outside the inclusion criteria; (2)The liver function was Child's class C and did not improved by medical treatment; (3)Infiltrative tumor which invaded and produced thrombosis of large vessels;(4)Lymphatic or distant metastasis.

#### Therapeutic instrument

The RA instrument (RFA-1000) was purchased from Beijing Bolaide, Inc. (Beijing, China). Low-power Sonostat was purchased from Jiangsu Hanmei Technology, Inc. (frequency: 20 kHz, power: ≤2 W, sound energy density: 0.63 W/cm^2^) (Jiangsu, China). Micro vesicle agent was: 10 mL of 5% sodium bicarbonate, 25 mL of 20% vitamin C, and 7.5 mL hydroxyethyl starch. The ultrasonograph instrument (iU22) was purchased from Philips, Inc. (Eindhoven, The Netherlands). Acoustic contrast agent was SonoVue (2.4 mL).

### Methods

This study was approved by the Ethics Committee of Kunming General Hospital. The subjects tested and their dependents provided their written informed consent to participate in this study. All clinical investigations were conducted according to the principles expressed in the Declaration of Helsinki. None of these 25 patients had received any anti-cancer treatment before the present study. An individualized RA plan was determined by a 3D conformal system using CT scan and contrast ultrasound. Ultrasound guidance was used during the RA procedure. One week after RA, and according to contrast ultrasound, LFUR was performed. The procedure was as follows: carbon dioxide microbubbles were injected through a peripheral vein in 60 seconds, and ultrasonic radiation treatment was immediately performed. Target zones were subject to ultrasonic radiation for 30 seconds. 5 minutes later, a second injection of microbubbles was given. Each patient underwent three courses of treatment, with five treatments per course and the interval between courses was two months. After treatment, patients received an IV drip with 250 mL 0.9% sodium chloride, 2.0 g etamsylate, and 0.2 g aminomethylbenzoic acid. LFUR was performed once a day during 6 days. After one day of rest, another 6 days of LFUR cycle was done. After RA/LFURM, patients with liver function of class B or C received drug treatment to ameliorate their liver function, and those without recurrence of cancer did not receive any anti-cancer treatment. For those with recurrence of cancer, hepatic resection and transcatheter arterial chemoembolization (TACE) were performed.

#### Evaluation of therapeutic effect

Contrast ultrasound, AFP and liver function tests were done before and 3, and 6 months after treatment. Quality of life (QOL) scores were applied before and 6 months after treatment. Results were graded as: good = 51∼60, improved = 41∼50, unchanged = 31∼40, and worst = 21∼30. An increase ≥10 in KPS scores was considered an improvement; a decrease ≥10 in KPS scores was considered worsening; while a range of +/−9 in KPS scores was considered unchanged.

#### Statistical analysis

Statistical analysis was performed using the Statistical Program for Social Sciences (SPSS Inc, Chicago, Illinois) version 19. Parametric variables were compared with paired T-test while non-parametric viariables were compared using the Chi-square test. Values of *P*<0.05 are considered significant.

## Results

### Decrease of gross tumor volumes

**Figure 1:**
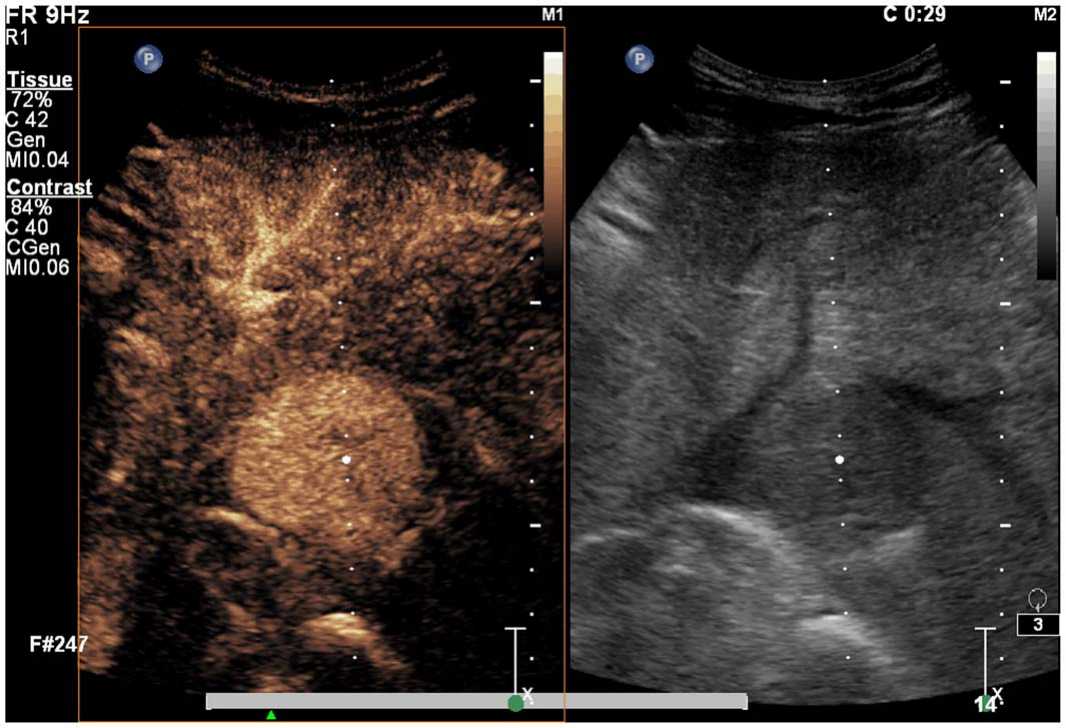
A spherical lesion with enhanced blood flow. Before treatment, a spherical lesion and enhanced blood flow is shown by contrast ultrasound.

**Figure 2:**
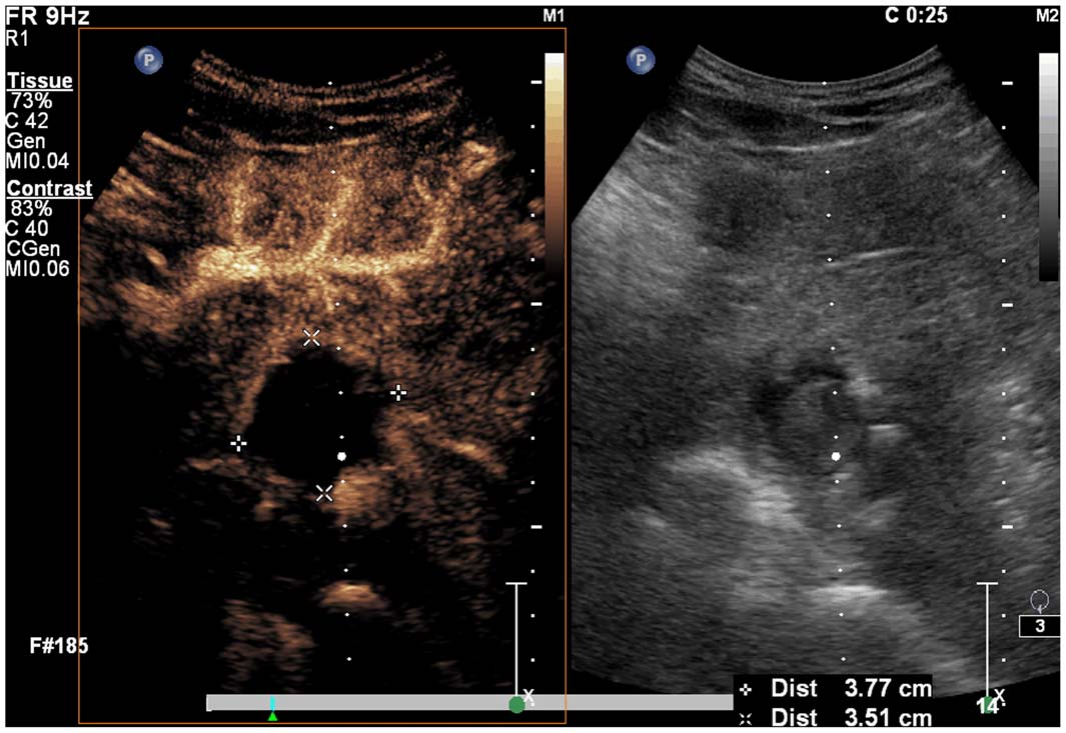
A spherical lesion with decreased volume and low blood flow. Three months after treatment the spherical lesion's volume decreased significantly, and there is no reinforcement of blood flow at the lesion with strengthening in tissues surrounding the lesion.

The gross tumor volumes ranged from 2.5 cm×2.0 cm to 5.5 cm×4.5 cm, as measured by contrast ultrasound 3 months after treatment in 16 patients. On average, the tumor volume decreased by approximately 30%. There was a significant decrease in tumor resonance. No increase in blood flow was found after treatment, but there was still blood supply to the tissues surrounding the tumor, which was shown by contrast ultrasound examination (Figure 1 A spherical lesion with enhanced blood flow)(Figure 2 A spherical lesion with decreased volume and low blood flow). Partial gross tumor volume decrease was found in 8 patients, 3 months after treatment. Minimal decrease of gross tumor volume was found in one patient.

**Figure 3:**
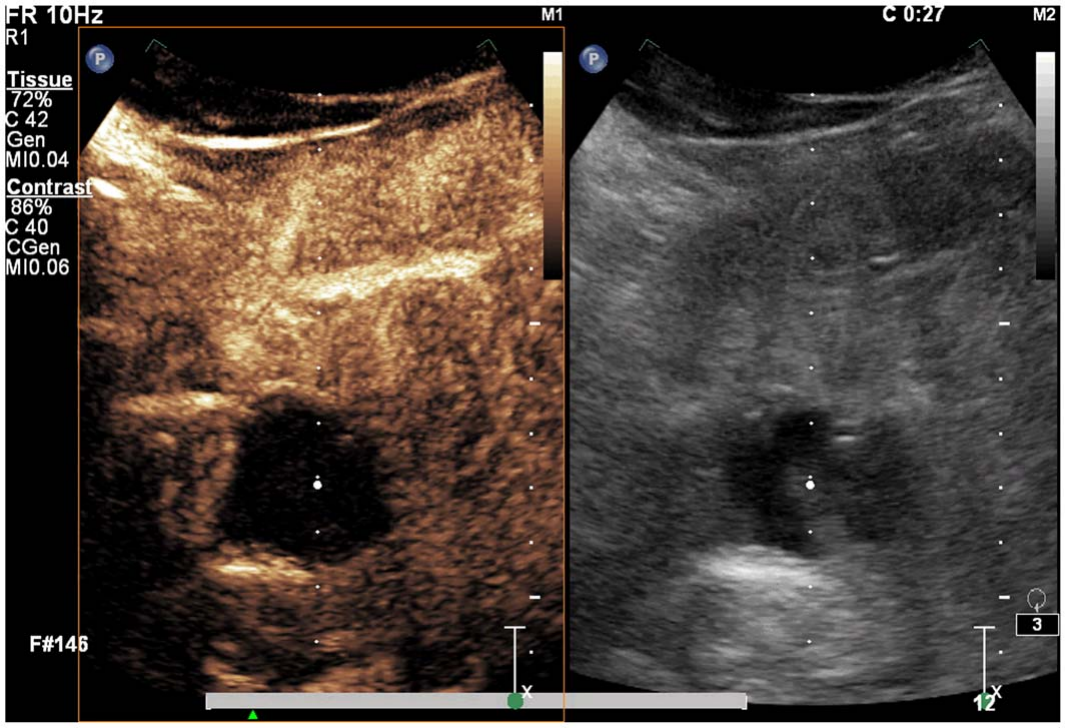
A spherical lesion with significantly decreased volume and no blood flow. Six months after treatment there is a significant decrease in the volume of the spherical lesion, with no reinforcement of its blood flow. There is no evident increase in blood flow of tissues surrounding the lesion.

After six months, the gross tumor volume decreased to 2.0 cm×1.8 cm∼4.9 cm×4.0 cm in 17 patients. Mean tumor volume decreased about 50% after treatment. There was a minimal blood flow increase during the arterial phase, portal venous phase, and delayed phase by contrast ultrasound. No blood flow was shown in tissue surrounding the resection area (Figure 3 A spherical lesion with significantly decreased volume and no blood flow). The *g*ross tumor volume decreased minimally, and blood flow enhanced significantly 3 months after treatment in one patient with CC who developed intrahepatic metastasis.

**Figure 4:**
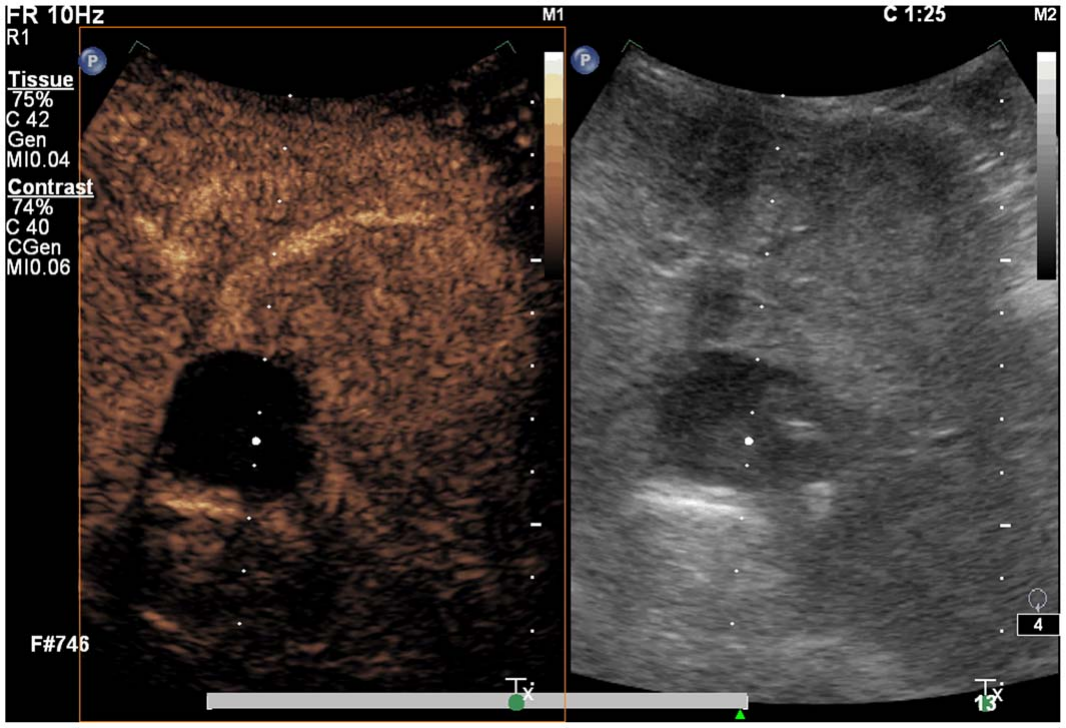
A spherical lesion with no blood flow. One year after treatment, the volume of the spherical lesion decreased mildly, but with no reinforcement of its blood flow. No blood flow was shown in the tissue surrounding the resection area.

After one year, the gross tumor volume decreased to 1.8 cm×1.7 cm∼4.7 cm×3.9 cm in 16 patients. The mean tumor volume decreased about 10% in the second 6 months than the first 6 months after treatment. A minimal blood flow increase was shown by contrast ultrasound during the arterial phase, portal venous phase, and delayed phase. No blood flow was shown in the tissue surrounding the resection area (Figure 4 A spherical lesion with no blood flow). The gross tumor volume decreased mildly and blood flow enhanced significantly one year after treatment in the other two patients who experienced cancer recurrence and intrahepatic metastasis, respectively.

### Liver function improvement and serum AFP decrease after treatment

In some patients ALT increased after treatment but the general liver function improved. There was a significant reduction of AST and TBIL 3 months after treatment (t = 23.51, 13.79, *P*<0.05). Albumin (Alb) levels increased 3 and 6 months after treatment but the differences were not significant (t = 1.76, 2.33, *P*>0.05). AFP decreased significantly after 3 and 6 months (t = 257.07, 333.46, *P*<0.05) (Table 1). At one year after RA/LFUR, compared with at 6 months after the treatment, ALT, AST, TBIL and Alb changed insignificantly, and AFP increased minimally (3.6±1.5 vs 7.2 ng/mL, t = 1.54, *P*>0.05) in a patient with CC who experienced cancer recurrence and increased significantly (6.9±2.4 vs 305.5±3.7 ng/mL, t = 51.36, *P*<0.05) in a patient with HCC who developed intrahepatic metastasis.

**Table 1:** Liver function and AFP values of 25 patients with malignant liver tumors before and after treatment with Radiofrequency Ablation combined with Low-Frequency Ultrasound Radiation with Microbubbles.

Group	n	ALT (IU/L)	AST (IU/L)	Alb (g/L)	TBIL (µmol/L)	AFP (ng/mL)
Pre-treatment	25	33.5±2.6	72.9±18.7	35.4±2.6	44.8±68.5	895.8±245.7
3 months after treatment	25	30.5±2.0^b^	55.3±11.5^a^	47.2±2.1^b^	16.4±5.8^a^	12.7±17.5^a^
6 months after treatment	25	17.4±2.0^a^	13.8±10.5^a^	49.1±2.0^b^	11.4±4.2^a^	7.4±2.4^a^
*1 year after treatment*	*25*	*16.5±1.8* b	*13.6±9.9* b	*50.3±1.9* b	*10.8±4.7* b	*8.6±3.2* b

Note:

a compared to pre-treatment, *P*<0.05;

b compared to pre-treatment, *P*>0.05.

### Follow-up

During the six months follow-up, none of the patients had tumor recurrence. QOL scores before treatment were good in 15 patients (60%), improved in 5 patients (20%), unchanged in 3 patients (12%) and worst in 2 patients (2/25, 8%). QOL scores post-treatment were good in 21 patients (84%), improved in 2 patients (8%), and unchanged in 1 patient (4%) and worst in 1 patient (4%). KPS for functional status for pre-treatment were improved in 16 patients (64%), stable in 7 patients (28%) and aggravated in 2 patients (8%). KPS post-treatment were improved in 19 patients (76%), stable in 5 patients (*20%*) and aggravated in 1 patient (4%). Difference between the pre-treatment and the post-treatment QOL and KPS were significant (χ^2^ = 41.600, 18.960, *P*<0.05). During the second six months of follow-up, there were no significant differences in the QOL scores and the KPS scores between at 6 months and at 1 year after treatment(χ^2^ = 39.7, 18.960, *P*<0.05). However, during 1-year follow-up, one of twenty-two patients with HCC developed intrahepatic metastasis (1/22, 4.5%), two of three patients with CC experienced cancer recurrence and intrahepatic metastasis (2/3, 66.7%), and there was a significant difference in the recurrence and metastasis rate at 1 year after treatment between the HCC and CC groups (χ^2^ = 10.388, *P*<0.011).

## Discussion

RA with a radiofrequency wave (460 KHZ) increases tissue and cell temperature (80∼100°C), resulting in the irreversible coagulation necrosis of the tumors and the liver parenchyma surrounding tumor [7]. RA is of limited use in tumors larger than 5 cm or in those of globular or irregular shape. RA destroys most tumor tissue and blood vessels, but leaves micro-tumor tissue and micro-vessels untouched in the periphery with the possibility of recurrence [8].

LFURM on the other hand produces ultrasonic cavitation affecting tumor micro-circulation. The microbubbles produce cavitation nuclei, which leads to extremely high shear stress force on the air bubble and deforms it until it fractures, with the resulting breakdown of cells and the bio macromolecules close to tumors, via the biological effect of ultrasonication. Moreover, the energy released during the cavitation process generates localized hyperthermia, hyperpiesia, shock wave, high speed micro- aspirating gas stream and free radicals [9].

Solid tumors generally contain abundant, poorly differentiated vessels, which are liable to destruction and develop embolism when subjected to ultrasonic cavitation. Low-frequency and low-power ultrasound radiation, when used in combination with intravenously injected microbubbles, can result in cavitation as efficiently as high power ultrasound radiation does. Hence, it may be a safe, minimally invasive treatment for tumors [10], [11].

Some research results have shown that the cavitation erosion induced by the low output power destroyed only the new vessels from tumor tissues, but had no impairment on normal tissues. However, the process of ischemia and necrosis of tumor tissues induced by cavitation with ultrasonic radiation microbubbles was slow. The large blood vessels supplying the tumor were not completely destroyed [12]. Other investigative results have also shown that low frequency ultrasound alone or combined with microbubbles can inhibit the proliferation of hepatocellular carcinoma cells and contribute to apoptosis, and that contribution to inducing apoptosis is more obvious when microbubbles are used [13].

Treatment by RA destroyed tumor cells. Treatment by LFURM attacks tumor micro-circulation complementing the effects of RA. So, the combined use by RA and LFURM could reach to the purpose of conformal treatment, reducing destruction of normal hepatic tissue, protecting liver function as much as possible, and increasing treatment efficacy.

The present study found a decrease in the gross volume of tumor with the combination of RA and LFURM. The transient increase of ALT in some patients suggests an effect of the treatment on normal hepatic tissue.However, the liver function recovered after treatment with usual liver protecting measures, suggesting that the impairment in liver function after treatment is reversible. Results suggest that the treatment can control local recurrence and increase patient survival. A randomized controlled study with long term follow-up is needed in order to prove this hypothesis.

The present study shows a significant difference in the recurrence and metastasis rate at 1 year after treatment between the HCC and CC groups, and such difference may correlate with the pathologic characteristics and biological behavior of CC. Patients with CC usually have no cirrhosis and do not develop choloplania, and their levels of AFP are normal. In addition, the early stage of CC development is insidious, which reduces the likelihood of early interventions. Consequently, the overall prognosis of CC is poor. Slakey et al [14]–[24] and others reported treatment of CC by RA in a limited number of cases, and they thought RA as a method to treat relapsing CC. Nevertheless, the therapeutic effect of RA and resection should be further compared in larger numbers of cases.
